# One-Year Safety and Effectiveness of Bivalirudin *versus* Heparin in Patients Undergoing Elective Percutaneous Coronary Intervention

**DOI:** 10.31083/j.rcm2408218

**Published:** 2023-07-31

**Authors:** Jiawen Li, Yulong Li, Shuhong Su, Zhifang Wang, Haiwei Liu, Weixian Yang, Shubin Qiao, Yuejin Yang, Bo Xu, Runlin Gao, Jinqing Yuan, Xueyan Zhao

**Affiliations:** ^1^Department of Cardiology, National Clinical Research Center for Cardiovascular Diseases, State Key Laboratory of Cardiovascular Disease, Fuwai Hospital, National Center for Cardiovascular Diseases, Chinese Academy of Medical Sciences and Peking Union Medical College, 100037 Beijing, China; ^2^Department of Cardiology, Xinxiang Central Hospital, 453000 Xinxiang, Henan, China; ^3^Department of Cardiology, Northern Theatre General Hospital, 110016 Shenyang, Liaoning, China

**Keywords:** bivalirudin, elective percutaneous coronary intervention, glycoprotein IIb/IIIa inhibitors, unfractionated heparin

## Abstract

**Background:**

Bivalirudin reduces ischemic and hemorrhagic events in patients undergoing primary percutaneous coronary intervention (PCI), but the safety and efficacy for such individuals are unclear. Our aim was to evaluate the long-term safety and efficacy of bivalirudin in patients undergoing elective PCI.

**Methods:**

We examined 957 patients with bivalirudin anticoagulation and 1713 patients with unfractionated heparin (UFH) anticoagulation with and without glycoprotein IIb/IIIa inhibitors (GPI). The primary endpoint was net adverse clinical events (NACE), a composite of death, myocardial infarction, revascularization, stent thrombosis, stroke, and bleeding. The secondary endpoints were bleeding and major adverse cardiovascular and cerebrovascular events (MACCE).

**Results:**

In one year of follow-up, 307 (11.5%) NACEs, 72 (2.7%) bleedings, and 249 (9.3%) MACCEs occurred. Statistically, patients with bivalirudin anticoagulation had less NACE [hazard ratio (HR): 0.75, 95% confidence interval (CI): 0.58–0.96, *p* = 0.021] and bleeding (HR: 0.58, 95% CI: 0.34–0.99, *p* = 0.045) but not less MACCE, than did those with UFH anticoagulation. Furthermore, the risk of bleeding in the bivalirudin group was lower than in the UFH with GPI group (*p* = 0.001) but not lower than in the group of UFH without GPI (*p* = 0.197).

**Conclusions:**

In patients who undergo elective PCI, the use of bivalirudin significantly decreased the risk of NACE and bleeding without increasing the risk of MACCE; the reduction of bleeding risk with bivalirudin was mainly attributed to the presence of GPIs in the UFH group.

## 1. Introduction

Percutaneous coronary intervention (PCI) has emerged as a major method to induce revascularization in patients with coronary heart disease. In a preparation for PCI, the use of intravenous anticoagulant treatment can effectively decrease the incidence of ischemic events [1, 2], but it may also raise the chances of bleeding [3]. The anticoagulant bivalirudin is a synthetic, reversible, and direct, thrombin inhibitor consisting of 20 amino acids. Its advantages lie in not activating, and not reducing the number of, platelets [4], so guidelines recommend it as an anticoagulant during PCI for patients with acute coronary syndrome (ACS). Previously, many international large-scale studies showed that bivalirudin treatment during primary PCI can reduce the incidence of bleeding events but may increase that of acute stent thrombosis (ST) more than does unfractionated heparin (UFH) [with or without glycoprotein IIb/IIIa receptor inhibitor (GPI)] [5, 6, 7, 8]. Nonetheless, the Bivalirudin in Acute Myocardial Infarction *vs*. Heparin and GPI Plus Heparin Trial (BRIGHT) has shown that in patients with acute myocardial infarction (MI) who underwent primary PCI, bivalirudin with a median 3-h post-procedure PCI-dose infusion decreased the risk of bleeding but did not raise that of ST and major adverse cardiovascular and cerebrovascular events (MACCEs) [9]. Out of concern for the risk of ST, the recommended grade of bivalirudin for patients with ACS has been revised, in several recent guidelines from class I [1, 10] to class IIa or class IIb [11, 12].

Recently, elective PCI has become more common. As bivalirudin has been demonstrated to have a lower risk of bleeding than does UFH [5, 6, 7, 8], some physicians choose to use bivalirudin in elective PCI patients as well [13], despite the fact that most of the literature on the efficacy and safety of bivalirudin is focused on primary PCI patient samples [5, 6, 7, 8, 9]. Moreover, the large-scale studies on patients with elective PCI were published over 10 years ago and lacked long-term bleeding evaluation [14, 15, 16, 17]. Additionally, with the rapid development of intervention techniques, the increase of complex lesions, and the use of a new generation of drug stents, that evidence has become obsolete and unrepresentative. International guidelines have not yet further updated and evaluated the recommended grade of bivalirudin for patients with elective PCI. The present study was intended to compare the one-year risk of ischemia and bleeding in elective PCI patients treated with either bivalirudin or UFH (with and without GPI), thereby bringing clinical data to the therapeutic strategy of patients with elective PCI.

## 2. Methods

### 2.1 Study Design

The study described herein was a prospective, multicenter, observational study. There were 1152 elective PCI patients, anticoagulated with bivalirudin, that were consecutively enrolled between January 2017 and August 2018 from 3 hospitals: Fuwai Hospital; Northern Theater General Hospital; and Xinxiang Central Hospital. Inclusion criteria included: (a) an age of 18–85 years; and (b) patients who were to undergo elective PCI. Exclusion criteria included: (a) primary PCI performed for ACS; and (b) ongoing warfarin or oral anticoagulant, with non-vitamin K antagonist, treatment. In addition, there were 10,250 patients that also met the criteria who underwent elective PCI and were anticoagulated with UFH (both with and without GPI), that were consecutively enrolled from January 2013 to December 2013 in Fu Wai Hospital. In this study, the patients receiving elective PCI include those with stable coronary heart disease and ACS patients who do not need emergency treatment, since patients who need emergency PCI have been excluded. The baseline characteristics of the total sample (*n *= 11,402) are shown in **Supplementary Table 1**. After 1:2 propensity-score matching (PSM), 2670 patients were eventually included in the study, among which there were 957 anticoagulated with bivalirudin, and 1713 anticoagulated with UFH (both with and without GPI).

### 2.2 Procedure and Medications

Patients routinely took aspirin and a P2Y12-receptor inhibitor (clopidogrel or ticagrelor) before the PCI procedure. Those who had not taken any P2Y12-receptor inhibitor previously were given a loading dose of either 300 mg of clopidogrel or 180 mg of ticagrelor before the procedure. After PCI, treatment with clopidogrel (75 mg, daily) or ticagrelor (90 mg, twice daily) was continued for at least 1 year.

For patients anticoagulated with bivalirudin, we administered an intravenous injection of bivalirudin (0.75 mg/kg) (Shenzhen Salubris Pharmaceuticals Co., Ltd., Shenzhen, China) before the PCI procedure, an intravenous infusion (1.75 ${\mathrm{mg/kg}}^{-1}$/$h^{-1}$) during PCI, and another treatment 3–4 h after PCI. The choice of bivalirudin was at the discretion of the physicians, and most chose it when there was a high risk of bleeding. For patients anticoagulated with UFH (either with or without GPI), we administered UFH (100 IU/kg) via artery-sheath catheter during PCI; an additional 1000 IU would be added if the PCI operation lasted longer than 1 h. After the activated coagulation time, physicians adjusted the dosage. The interventional cardiologist determined whether to employ GPI based on the clinical circumstances and coronary lesions during the procedure.

### 2.3 Definitions and Outcomes

The definition of bleeding used was the Bleeding Academic Research Consortium (BARC) criteria type 2, 3, or 5 bleeding [18]. The definition of MACCE used was a composite of death, MI, revascularization, ST, and stroke. The definition of net adverse clinical events (NACE) used was a composite of bleeding and MACCE.

The primary endpoint was NACE. The secondary endpoints were bleeding and MACCE. A follow-up evaluation of patients was done one year after discharge. Follow-up data were collected by an independent team of clinical physicians through clinic visits, phone interviews, or texts. Endpoint events were judged by two independent cardiologists, and disagreements were settled by their consensuses.

### 2.4 Statistical Analysis

A thorough explanation of the statistical methods is provided in the online **Supplementary Material**. The PSM was used to identify the patients in the two groups (bivalirudin and UFH) with similar baseline characteristics. Univariate and multivariate Cox regressions were conducted to calculate the hazard ratio (HR) and 95% confidence interval (CI), and evaluate the associations between bivalirudin and clinical outcomes. Statistical significance was defined as two-tailed *p <* 0.05. PSM was conducted using R software v. 3.4.3 (R Core Team, Vienna, Austria). The other analyses were performed using SPSS software v. 23.0 (IBM Corp., Armonk, NY, USA).

## 3. Results

**Fig. 1.**
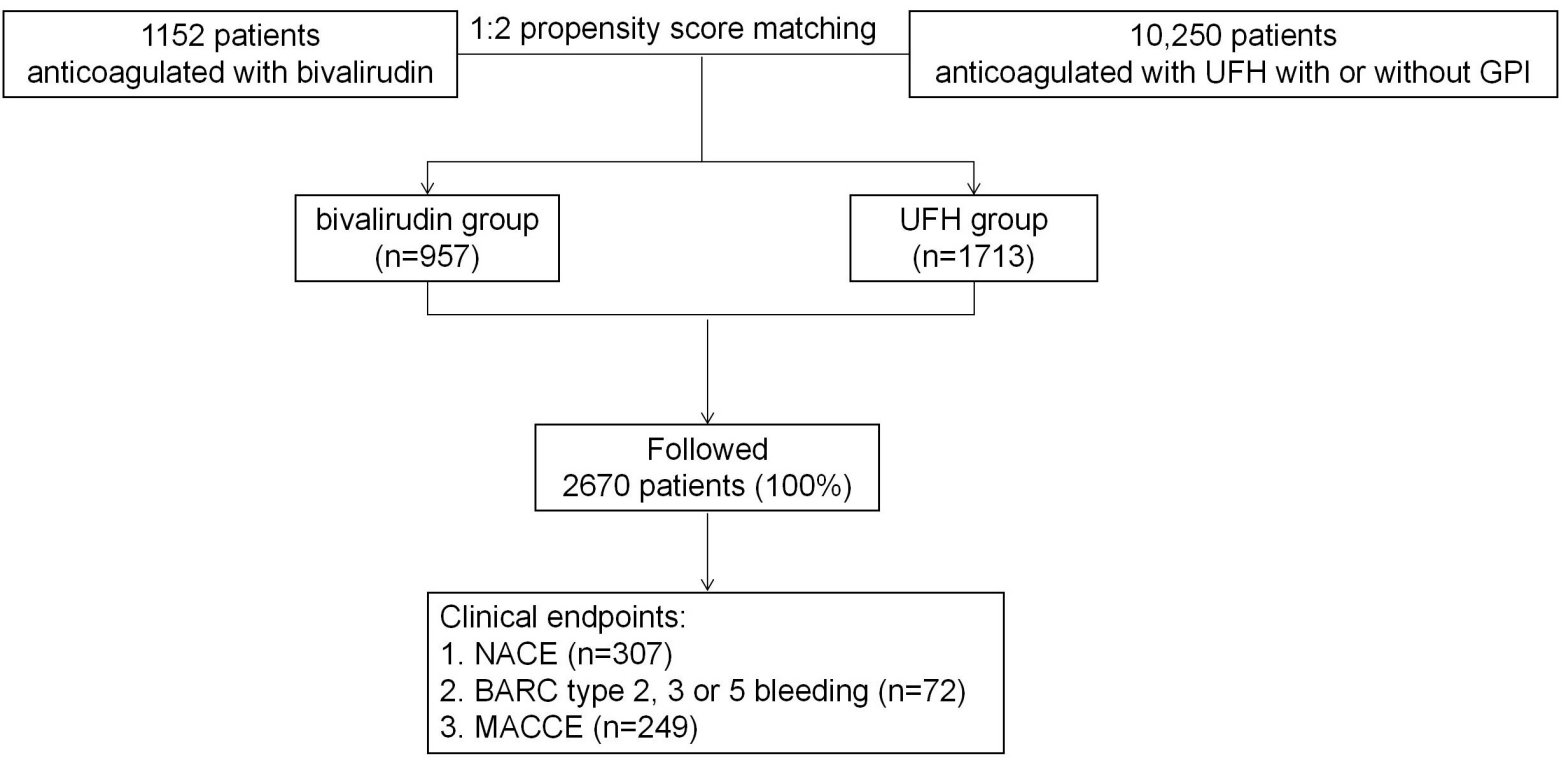
**Flow chart**. BARC, Bleeding Academic Research Consortium; UFH, unfractionated heparin; GPI, glycoprotein IIb/IIIa inhibitors; MACCE, major adverse cardiac and cerebrovascular events; NACE, net adverse clinical events.

The resulting 2670 patients were included after 1:2 PSM (Fig. 1). During long-term follow-up (median follow-up = 1.08 years) with a response rate of 100%, there were 307 (11.5%) NACE incidents, 72 (2.7%) bleeding incidents, and 249 (9.3%) MACCE incidents.

### 3.1 Baseline Characteristics

Among the 2670 patients, the mean (SD) age was 68.15 (9.19) years, and 1814 (67.9%) were male. The mean (SD) age of the 957 patients in the bivalirudin group was 68.43 (10.04) years, and that of the 1713 patients in the UFH group was 68.00 (8.67) years. In the UFH group, 254 (15.0%) patients also were treated with GPI. In the bivalirudin group, patients meeting major criteria for the Academic Research Consortium for High Bleeding Risk (ARC-HBR) [19] were: 37 (3.9%) hemoglobin <11 g/dL, and 6 (0.6%) moderate or severe baseline thrombocytopenia (platelet count <100 × ${\mathrm{10}}^{9}$/L). Patients meeting minor criteria for the ARC-HBR were as follows: 297 (31.0%) were age ≥75 years, 117 (12.2%) had hemoglobin of 11–12.9 g/dL for men and 11–11.9 g/dL for women, and 255 (26.7%) had prior stroke.

The baseline characteristics of the bivalirudin and UFH groups are shown in Table 1. Patients in the bivalirudin group had a lower level of white blood cell count (WBC), a higher level of high-sensitivity C reactive protein, a lower incidence of prescribed clopidogrel, a lower incidence of chronic obstructive pulmonary diseases, greater smoking history, more prior incidences of MI and PCI, and fewer prior incidences of coronary artery bypass grafting, than did the patients in the UFH group.

**Table 1.** **Baseline Characteristic**.

Parameters		Bivalirudin (*n* = 957)	UFH (*n* = 1713)	*p* value
Demographic characteristics				
	Age (years)	68.43 ± 10.04	68.00 ± 8.67	0.260
	Male	646 (67.5)	1168 (68.2)	0.717
	Body mass index (${\mathrm{kg/m}}^{2}$)	25.60 ± 9.84	25.42 ± 3.14	0.583
Cardiovascular risk factor				
	Diabetes	385 (40.2)	671 (39.2)	0.592
	Hypertension	693 (72.4)	1261 (73.6)	0.502
	Hyperlipidemia	707 (73.9)	1236 (72.2)	0.338
	Chronic obstructive pulmonary disease	9 (0.8)	78 (4.6)	<0.001
	Peripheral vascular disease	80 (8.4)	129 (7.5)	0.445
	Current/former smoker	536 (56.0)	833 (48.6)	<0.001
	Previous myocardial infarction	250 (26.1)	340 (19.8)	<0.001
	Previous percutaneous coronary intervention	268 (28.0)	419 (24.5)	0.045
	Previous coronary artery bypass grafting	29 (3.0)	94 (5.5)	0.004
	Previous cerebrovascular disease	255 (26.6)	440 (25.7)	0.588
	Family history of coronary heart disease	121 (12.6)	226 (13.2)	0.686
Laboratory results at admission				
	Hemoglobin (g/dL)	14.13 ± 1.74	14.14 ± 1.60	0.916
	White blood cell count (${\mathrm{10}}^{9}$/L)	6.66 ± 1.80	6.97 ± 1.88	<0.001
	Platelet count (${\mathrm{10}}^{9}$/L)	224.09 ± 62.14	221.57 ± 63.34	0.321
	Low-density lipoprotein cholesterol (mmol/L)	2.36 ± 0.82	2.38 ± 0.94	0.689
	High-sensitivity C reactive protein (mg/L)	2.10 (0.91, 3.13)	1.63 (0.80, 3.59)	0.007
	Left ventricular ejection fraction (%)	60.70 ± 6.84	60.36 ± 13.43	0.381
Medication				
	Clopidogrel	921 (96.2)	1693 (98.8)	<0.001
	Glycoprotein IIb/IIIa inhibitors	-	254 (15.0)	-

Values are mean ± standard deviation or *n* (%). UFH, unfractionated heparin.

### 3.2 Incidence Rates of Clinical Outcomes

The incidence rate of the primary endpoint, NACE, in the bivalirudin group was lower than that in the UFH group [89 (9.3%)* vs.* 218 (12.7%), *p* = 0.008)].

The incidence rates of bleeding and MACCE in the bivalirudin group were similar to those in the UFH group [18 (1.9%) *vs.* 54 (3.2%), *p* = 0.052 for bleeding; 77 (8.0%) *vs.* 172 (10.0%), *p* = 0.089 for MACCE].

### 3.3 One-Year Clinical Outcomes of Bivalirudin and UFH Groups

*NACE*. In the univariate Cox model, WBC, low-density lipoprotein cholesterol (LDL-C), and peripheral vascular disease (PVD) were associated with NACE (**Supplementary Table 2**). Multivariate analysis showed that bivalirudin decreased the risk of NACE (adjusted HR: 0.75, 95% CI: 0.58–0.96; *p *= 0.021) more than did UFH (with or without GPI) (Table 2).

**Table 2.** **One-year outcomes of bivalirudin and UFH**.

Outcomes	Anticoagulant during elective PCI	Events (%)	Crude HR (95% CI)	*p* value	Adjusted HR (95% CI)	*p* value
Primary endpoint						
NACE	UFH	218 (12.7)	–	–	–	–
	Bivalirudin	89 (9.3)	0.73 (0.57–0.93)	0.012	0.75 (0.58–0.96)	0.021
Secondary endpoints						
Bleeding	UFH	54 (3.2)	–	–	–	–
	Bivalirudin	18 (1.9)	0.60 (0.35–1.02)	0.058	0.58 (0.34–0.99)	0.045
MACCE	UFH	172 (10.0)	–	–	–	–
	Bivalirudin	77 (8.0)	0.80 (0.61–1.05)	0.107	0.82 (0.63–1.08)	0.159

PCI, percutaneous coronary intervention; UFH, unfractionated heparin; NACE, net adverse clinical events; MACCE, major adverse cardiac and cerebrovascular events; HR, hazard ratio; CI, confidence interval.

*Bleeding. *In the univariate Cox model, age and hemoglobin level were associated with bleeding risk (**Supplementary Table 2**). Multivariate analysis showed that bivalirudin decreased the risk of bleeding (adjusted HR: 0.58, 95% CI: 0.34–0.99; *p* = 0.045) more than did UFH (with or without GPI) (Table 2).

*MACCE. *In the univariate Cox model, PVD, WBC, platelet count, and LDL-C were associated with MACCE (**Supplementary Table 2**). Multivariate analysis showed that the risk of MACCE with bivalirudin treatment was comparable to that of UFH (with or without GPI) (adjusted HR: 0.82, 95% CI: 0.63–1.08; *p* = 0.159) (Table 2).

### 3.4 Subgroup Analyses for Bleeding 

**Fig. 2.**
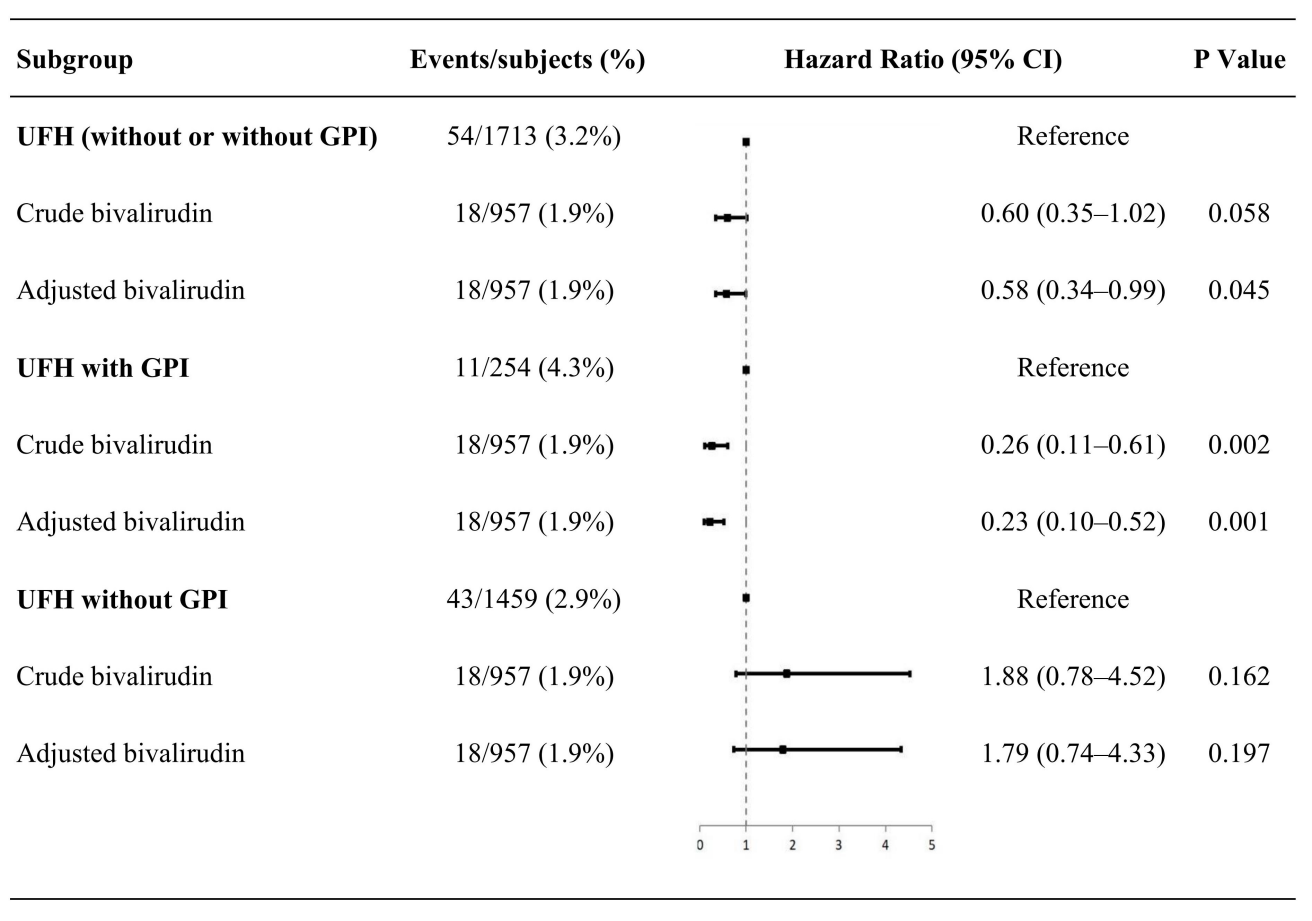
**Subgroup analyses of bleeding**. UFH, unfractionated heparin; GPI, glycoprotein IIb/IIIa inhibitors; CI, confidence interval.

*UFH with GPI. *Univariable and multivariable analyses showed that anticoagulation with bivalirudin was associated with a lower risk of bleeding (adjusted HR: 0.23, 95% CI: 0.10–0.52; *p* = 0.001) (Fig. 2) than was anticoagulation with UFH with GPI (*n *= 254).

*UFH without GPI. *There was no difference in the risk of bleeding between patients anticoagulated with UFH without GPI (*n *= 1459) and patients anticoagulated with bivalirudin (univariable and multivariable analyses, *p* > 0.05) (Fig. 2).

*One-year BARC type 3 or 5 bleeding of bivalirudin and UFH groups*. For further analysis, multivariate analysis adjusted by age, hemoglobin level, and PVD significant in univariate Cox model, showed that the risk of bleeding, defined by BARC type 3 or 5 bleeding, in the bivalirudin group was comparable to that in the UFH group (with or without GPI) (adjusted HR 1.70, 95% CI: 0.74–3.93; *p* = 0.214) (**Supplementary Table 3**).

## 4. Discussion

In this prospective, multicenter, observational study among patients with elective PCI, the main findings were as follows: (1) patients receiving bivalirudin treatment during PCI showed a lower NACE and bleeding risks with no elevated MACCE risk, than did patients receiving UFH (with or without GPI) treatment; (2) a further subgroup analysis showed that bivalirudin produced a lower risk of bleeding than did UFH with GPI but not than UFH without GPI; (3) bivalirudin during elective PCI decreased the risk of bleeding better in BARC type 2, 3, or 5 bleeding but not in BARC type 3 or 5 bleeding more than did UFH (with or without GPI).

### 4.1 NACE with Bivalirudin vs. UFH

Apparently, there have been few studies on bivalirudin in patients with elective PCI. Mostly, previous studies, which focused on patients with primary PCI, showed that bivalirudin could significantly reduce NACE risk compared with UFH [5, 6, 7, 8, 9]. The present study demonstrated that bivalirudin significantly decreased the risk of long-term (one year) NACE more than did UFH in patients undergoing elective PCI. Previous related studies involving mainly patients with elective PCI focused on short-term outcomes and their results were inconsistent. Tavano *et al*. [20] and the Acute Catheterization and Urgent Intervention Triage Strategy (ACUITY) trial [17] showed, consistent with our results, that bivalirudin, more than UFH with GPI, could significantly decrease 30-day NACE in diabetic patients with elective PCI (*n* = 335) and in patients with non-ST-segment-elevation ACS (NSTE-ACS). However, some large short-term studies, in which the proportion of patients undergoing elective PCI was high, were inconsistent with our results. The Randomized Evaluation in PCI Linking Angiomax to Reduced Clinical Events (REPLACE)-2 trial [14] showed that in patients with urgent or elective PCI (*n* = 6010), no significantly different 30-day NACE was observed between patients anticoagulated with bivalirudin and those anticoagulated with UFH. The Intracoronary Stenting and Antithrombotic Regimen: Rapid Early Action for Coronary Treatment (ISAR-REACT) 3 trial [15] showed that for 30-day NACE, no significant difference was observed between the bivalirudin group and the UFH group in patients with stable or unstable angina undergoing PCI (*n* = 4570). In the ISAR-REACT 4 trial [16], for 30-day NACE, no significant difference was found between the bivalirudin group and UFH plus abciximab group in patients with non-ST-segment elevation MI undergoing PCI (*n* = 1721). Of note, these studies [14, 15, 16] (a) did not entirely focus on patients with elective PCI; (b) patients were followed up for only 30 days and (c) BARC criteria were not adopted to define bleeding events.

### 4.2 Ischemic Events with Bivalirudin vs. UFH

Some study results on primary PCI suggested that acute ST events may be related to the short half-life of bivalirudin [5, 6, 7, 8], so premature discontinuation of bivalirudin after PCI may increase the risk of ST. The large BRIGHT trial [9], in Chinese patients with primary PCI, established that the use of bivalirudin with prolonged infusion (for 3–4 h after PCI) did not lead to increased ST risk, and an unrelated study [21] confirmed that prolonging infusion of bivalirudin after primary PCI is a promising method of treatment. Therefore, we adopted this method in the elective PCI population, and surprisingly no increased incidence of MACCE was observed.

Previous articles on elective PCI showed that bivalirudin treatment and UFH treatment result in similar rates of ischemic events. Bangalore *et al*. [22] conducted a registry study in patients with NSTE-ACS and with stable ischemic heart disease (*n* = 1036) and found that UFH alone and bivalirudin had similar incidences of in-hospital and one-year ischemic events. Tavano *et al*. [20] found that in diabetic patients with elective PCI (*n *= 335), UFH plus tirofiban treatment and bivalirudin treatment had similar incidences of 30-day ischemic events. High-quality trials, including ACUITY [17, 23], REPLACE-2 [14, 24], ISAR-REACT-3 [15, 25], ISAR-REACT-4 [16, 26], and Novel Approaches in Preventing and Limiting Events III Trial: Bivalirudin in High-Risk Bleeding Patients (NAPLES-Ⅲ) [27], consistently demonstrated that bivalirudin was comparable to UFH regarding 30-day and one-year ischemic events. The above results were consistent with ours, indicating anticoagulation with bivalirudin during elective PCI does not increase the risk of ischemic events, and further provide effective experimentally-based evidence for the use of bivalirudin in elective PCI.

As for the comparison of the one-year all-cause death rate between the two anticoagulants used, we found that the rate was comparable in the two groups [bivalirudin: 13 (1.4%) *vs*. UFH: 20 (1.2%), *p* = 0.669]. A previous meta-analysis examining four randomized, controlled trials, demonstrated that in patients with acute ST-elevation myocardial infarction (STEMI) undergoing primary PCI, the one-year all-cause death rate was lower in patients anticoagulated with bivalirudin than in those with anticoagulated with UFH plus GPI [28], which was inconsistent with our results. The one-year all-cause death reduction in that study was hypothesized to be more likely due to a reduced iatrogenic haemorrhagic complication. It is of note that the subjects we enrolled were all patients with elective PCI. The subjects enrolled in the meta-analysis study were all patients with STEMI who are fragile, and it seems that major bleeding, through various pathophysiological mechanisms, may have destabilized an already unstable condition [29].

### 4.3 Hemorrhagic Events with Bivalirudin vs. UFH

Most patients in this study were considered by physicians to be at high risk of bleeding. Nonetheless, bivalirudin still showed a reduction in the incidence of one-year bleeding in these patients more than did UFH. In patients with elective PCI, there were few studies that compared bleeding in bivalirudin and UFH, and the results of those studies were inconsistent.

Studies by Bangalore *et al*. [22], Tavano *et al*. [20], and the ACUITY [17], REPLACE-2 [14], ISAR-REACT-3 [15], and ISAR-REACT-4 [16] trials, demonstrated that bivalirudin could decrease 30-day bleeding significantly more than did UFH (consistent with our result). However, the NAPLES-Ⅲ trial [27] showed that according to several different criteria, there was no difference in the rate of in-hospital bleeding between bivalirudin and UFH in patients with elective PCI and high bleeding risk (*n *= 837), and that the risk of in-hospital hemorrhage was comparable in the two groups. This differed from our results; the possible explanation may be that the low dose of UFH (70 IU/kg) without a GPI regimen in their study [27] made the major bleeding rate in patients anticoagulated with UFH lower than anticipated. Hence, there is controversy over the association of bivalirudin and UFH with bleeding in patients with elective PCI. It is worth mentioning that the above results regarding bleeding were all from short-term studies without a long-term comparison. The present study fills that gap and further showed that anticoagulation with bivalirudin during elective PCI could result in a reduction in long-term bleeding.

At present, we believe that the significant difference in bleeding between bivalirudin and UFH may be attributable to the inclusion of GPIs in the UFH group, because the incidence of bleeding is comparable in the two groups of patients with primary PCI when a comparable GPI was used [30]. To help clarify this issue, we conducted a subgroup analysis, in which, there were 254 (15%) patients anticoagulated with UFH with GPI and 957 (100%) patients anticoagulated with bivalirudin without GPI. Subgroup analysis showed that UFH with GPI reduced the risk of bleeding less than did bivalirudin, but UFH without GPI and bivalirudin had comparable bleeding risks. This finding further supported the opinion that the statistically significant difference resulted from the additional use of GPIs in the UFH group. We showed that in China, when using UFH, the frequency of GPI use is relatively high in elective procedures, although the use of GPI was not recommended routinely.

The present study provided real-world evidence for recommending bivalirudin in patients with elective PCI. In such patients, a comparison was made between bivalirudin (with 3–4 h post-procedure PCI-dose infusion) with UFH in terms of safety and effectiveness. After PSM and multivariate adjustment, we found that (a) the risk of bleeding was significantly reduced by bivalirudin without increasing the risk of MACCE, and (b) the reduction of bleeding risk in UFH-treated patients was mainly attributable to the additional use of GPIs.

### 4.4 Limitations

First, this real-world study was observational with intrinsic defects; such an observational study cannot establish a cause-effect relationship. Second, this study included only Chinese patients, and the effects on different races and ethnicities need to be analyzed in the future. Third, this research lacked data from a UFH group collected from the same time period as the bivalirudin group. Although we have tried our best to remove that bias, there may still be some deviations that cannot be avoided. Fourth, although many patients were screened and PSM and multivariate adjustment were used, residual confounding factors may not have been completely eliminated. Fifth, the sample size was still small, so that the rate of outcomes after 30 days was too low to produce a reliable short-term result. In the future, it is worth carrying out the study on a larger sample in order to compare the short-term effectiveness and safety of bivalirudin and UFH in this kind of patient.

## 5. Conclusions

In patients with elective PCI, bivalirudin during PCI significantly reduced the risk of NACE and bleeding. The risk of bleeding was significantly lower in patients anticoagulated with bivalirudin than in those with UFH, which may be associated with the combined use of GPIs in the latter. The present study provided reliable real-world clinical data for the use of bivalirudin in patients with elective PCI.

## Data Availability

Due to ethical restrictions related to the consent given by subjects at the time of study commencement, our datasets are available from the corresponding author upon reasonable request after permission of the Institutional Review Board of State Key Laboratory of Cardiovascular Disease, Fuwai Hospital, National Centre for Cardiovascular Diseases.

## Abbreviations

PCI, percutaneous coronary intervention; ACS, acute coronary syndrome; UFH, unfractionated heparin; GPI, glycoprotein IIb/IIIa receptor inhibitor; MI, myocardial infarction; BARC, Bleeding Academic Research Consortium; MACCE, major adverse cardiovascular and cerebrovascular events; NACE, net adverse clinical events; ST, stent thrombosis; HR, hazard ratio; CI, confidence interval; ARC-HBR, Academic Research Consortium for High Bleeding Risk; WBC, white blood cell count; LDL-C, low-density lipoprotein cholesterol; PVD, peripheral vascular disease; NSTE-ACS, non-ST-segment-elevation acute coronary syndrome; STEMI, ST-elevation myocardial infarction.

## Supplementary Material

Supplementary material associated with this article can be found, in the online version, at https://doi.org/10.31083/j.rcm2408218.
