# Supplementary material for: One-Year Safety and Effectiveness of Bivalirudin versus Heparin in Patients Undergoing Elective Percutaneous Coronary Intervention
Source: Rev Cardiovasc Med. 2023 Jul 31;24(8):218. doi: 10.31083/j.rcm2408218 (PMC11262434; doi:10.31083/j.rcm2408218)
Supplement: Supplementary file 1 [file 2153-8174-24-8-218-s1.docx]

**Supplementary Materials**

Li et al. One-Year Safety and Effectiveness of Bivalirudin versus Heparin in Patients Undergoing Elective Percutaneous Coronary Intervention

**Contents**

**Ⅰ. Supplementary Methods**

Statistical Analysis

**Ⅱ. Supplementary Tables**

**Table S1.** Baseline characteristic before propensity score matching analysis

**Table S2.** Univariate Cox regression of variables with clinical outcomes

**Table S3.** One-year BARC type 3 or 5 bleeding of bivalirudin versus UFH

**Ⅰ. Supplementary Methods**

Statistical Analysis

Continuous variables with a normal distribution were expressed as mean ± standard deviation, and continuous variables with non-normally distribution were expressed as median with quartiles. Categorical variables were expressed as numbers (%). Student’s *t*-tests or Mann-Whitney *U* tests were used to compare continuous variables while Chi-square tests were applied to compare categorical variables between the two groups. The patients in the 2 groups were matched with 17 significant variables of baseline characteristics [age, male, diabetes, hypertension, hyperlipidemia, chronic obstructive pulmonary disease, peripheral vascular disease (PVD), previous MI, previous PCI, previous cerebrovascular disease, family history of coronary heart disease, hemoglobin, platelet count, low-density lipoprotein cholesterol (LDL-C), high-sensitivity C reactive protein (hs-CRP), left ventricular ejection fraction and clopidogrel] in a 1:2 nearest neighbor manner with a caliper width equal to 0.02. All multivariate models were adjusted by the variables that showed significance in the corresponding univariate analysis. The multivariate model was adjusted for white blood cell count (WBC), LDL-C and PVD when endpoint was NACE. The multivariate model was adjusted for age and hemoglobin when endpoint was bleeding. The multivariate model was adjusted for WBC, platelet count, LDL-C and PVD when endpoint was MACCE. As for subgroup analysis, the multivariate model was adjusted for age and hemoglobin when compared with UFH (with or without GPI). The multivariate model was adjusted for age and PVD when compared with UFH with GPI. The multivariate model was adjusted for age, hemoglobin, and PVD when compared with UFH without GPI.

**Ⅱ. Supplementary Tables**

**Table S1.** Baseline characteristic before propensity score matching analysis

| Parameters | | Bivalirudin  (n = 1152) | UFH  (n = 10,250) | *p* value |
| --- | --- | --- | --- | --- |
| Demographic characteristics | |  |  |  |
|  | Age (years) | 68.60 ± 10.07 | 58.33 ± 10.23 | <0.001* |
|  | Male | 781 (67.8) | 7899 (77.1) | <0.001* |
|  | Body mass index (kg/m^2^) | 25.85 ± 14.07 | 25.92 ± 3.18 | 0.864 |
| Cardiovascular risk factor | |  |  |  |
|  | Diabetes | 472 (41.0) | 3111 (30.4) | <0.001* |
|  | Hypertension | 832 (72.2) | 6605 (64.4) | <0.001* |
|  | Hyperlipidemia | 863 (74.9) | 6917 (67.5) | <0.001* |
|  | Chronic obstructive pulmonary disease | 11(1.0) | 239 (2.3) | 0.002* |
|  | Peripheral vascular disease | 113 (9.8) | 227 (2.7) | <0.001* |
|  | Current/former smoker | 650 (56.4) | 5957 (58.1) | 0.270 |
|  | Previous myocardial infarction | 325 (28.2) | 1999 (19.5) | <0.001* |
|  | Previous percutaneous coronary intervention | 331 (28.7) | 2383 (23.2) | <0.001* |
|  | Previous coronary artery bypass grafting | 36 (3.1) | 434 (4.2) | 0.073 |
|  | Previous cerebrovascular disease | 313 (27.2) | 1089 (10.6) | <0.001* |
|  | Family history of coronary heart disease | 137 (11.9) | 2526 (24.6) | <0.001* |
| Laboratory results at admission | |  |  |  |
|  | Hemoglobin (g/dL) | 14.17 ± 1.71 | 14.30 ± 1.53 | 0.012* |
|  | White blood cell count (10^9^/L) | 6.74 ± 1.88 | 6.74 ± 1.80 | 0.946 |
|  | Platelet count (10^9^/L) | 228.86 ± 65.86 | 204.96 ± 54.36 | <0.001* |
|  | Low-density lipoprotein cholesterol (mmol/L) | 2.36 ± 0.81 | 2.43 ± 0.98 | 0.014* |
|  | High-sensitivity C reactive protein (mg/L) | 2.09 (0.91, 3.17) | 1.50 (0.73, 3.35) | <0.001* |
|  | Left ventricular ejection fraction (%) | 60.56 ± 6.92 | 61.65 ± 11.69 | <0.001* |
| Medication | |  |  |  |
|  | Clopidogrel | 982 (85.2) | 10,229 (99.8) | <0.001* |
|  | Glycoprotein IIb/IIIa inhibitors | - | 1636 (16.0) | - |

Values are mean ± standard deviation or n (%). UFH: unfractionated heparin.

*P values indicating statistical significance.

**Table S2.** Univariate Cox regression of variables with clinical outcomes

| Parameters | NACE | | Bleeding | | MACCE | |
| --- | --- | --- | --- | --- | --- | --- |
|  | HR (95% CI) | *p* value | HR (95% CI) | *p* value | HR (95% CI) | *p* value |
| Age | 1.00 (0.99–1.01) | 0.929 | 1.03 (1.01–1.06) | 0.022* | 0.99 (0.98–1.01) | 0.403 |
| Male | 0.99 (0.78–1.26) | 0.962 | 0.69 (0.43–1.11) | 0.128 | 1.09 (0.83–1.43) | 0.535 |
| Body mass index | 1.00 (0.99–1.02) | 0.917 | 0.97 (0.91–1.04) | 0.395 | 1.00 (0.99–1.02) | 0.777 |
| Diabetes | 1.13 (0.91–1.42) | 0.282 | 0.76 (0.47–1.24) | 0.276 | 1.22 (0.95–1.57) | 0.118 |
| Hypertension | 0.97 (0.76–1.25) | 0.835 | 1.10 (0.65–1.88) | 0.722 | 0.93 (0.71–1.23) | 0.628 |
| Hyperlipidemia | 0.99 (0;77–1.27) | 0.910 | 1.21 (0.70–2.09) | 0.490 | 0.93 (0.70–1.22) | 0.591 |
| Chronic obstructive pulmonary disease | 1.24 (0.70–2.20) | 0.470 | 0.86 (0.21–3.50) | 0.832 | 1.27 (0.67–2.39) | 0.461 |
| Peripheral vascular disease | 1.81 (1.30–2.53) | <0.001* | 1.92 (0.98–3.73) | 0.057 | 1.78 (1.23–2.57) | 0.002* |
| Current/former smoker | 1.15 (0.92–1.44) | 0.215 | 0.95 (0.60–1.51) | 0.828 | 1.19 (0.92–1.52) | 0.182 |
| Previous myocardial infarction | 0.99 (1.01–0.76) | 0.942 | 1.27 (0.75–2.15) | 0.367 | 0.96 (0.71–1.29) | 0.768 |
| Previous percutaneous coronary intervention | 1.03 (0.80–1.33) | 0.794 | 0.97 (0.57–1.65) | 0.897 | 1.08 (0.82–1.43) | 0.570 |
| Previous coronary artery bypass grafting | 1.14 (0.69–1.89) | 0.602 | 0.59 (0.15–2.42) | 0.466 | 1.44 (0.87–2.38) | 0.162 |
| Previous cerebrovascular disease | 1.13 (0.88–1.45) | 0.340 | 1.62 (1.00–2.61) | 0.051 | 0.98 (0.74–1.30) | 0.887 |
| Family history of coronary heart disease | 0.83 (0.58–1.19) | 0.308 | 0.72 (0.33–1.57) | 0.408 | 0.92 (0.63–1.34) | 0.657 |
| Hemoglobin | 0.99 (0.99–1.00) | 0.093 | 0.98 (0.96–0.99) | <0.001* | 1.00 (0.99–1.01) | 0.883 |
| White blood cell count | 1.10 (1.04–1.16) | <0.001* | 0.96 (0.84–1.09) | 0.503 | 1.13 (1.07–1.20) | <0.001* |
| Platelet count | 1.00 (1.00–1.00) | 0.093 | 1.00 (1.00–1.00) | 0.651 | 1.00 (1.00–1.00) | 0.036* |
| Low-density lipoprotein cholesterol | 0.86 (0.76–0.98) | 0.023* | 0.83 (0.64–1.09) | 0.184 | 0.86 (0.74–0.99) | 0.039* |
| High-sensitivity C reactive protein | 1.01 (0.98–1.05) | 0.397 | 0.99 (0.92–1.06) | 0.759 | 1.02 (0.99–1.06) | 0.157 |
| Left ventricular ejection fraction | 1.00 (0.99–1.01) | 0.817 | 1.02 (0.99–1.04) | 0.237 | 1.00 (0.99–1.01) | 0.607 |
| Clopidogrel | 1.08 (0.48–2.43) | 0.845 | 1.54 (0.21–11.06) | 0.670 | 1.05 (0.43–2.54) | 0.919 |

* P values indicating statistical significance.

NACE: net adverse clinical events; MACCE: major adverse cardiac and cerebrovascular events; HR: hazard ratio; CI: confidence interval.

**Table S3.** One-year BARC type 3 or 5 bleeding of bivalirudin versus UFH

| Outcomes | Anticoagulant during elective PCI | Events (%) | Crude HR (95% CI) | P value | Adjusted HR (95% CI) | P value |
| --- | --- | --- | --- | --- | --- | --- |
| BARC type 3 or 5 bleeding | UFH | 11 (0.6) | – | – | – | – |
|  | Bivalirudin | 12 (1.1) | 1.80 (0.78–4.15) | 0.168 | 1.70 (0.74–3.93) | 0.214 |

BARC: Bleeding Academic Research Consortium; PCI: percutaneous coronary intervention; HR: hazard ratio; CI: confidence interval.
